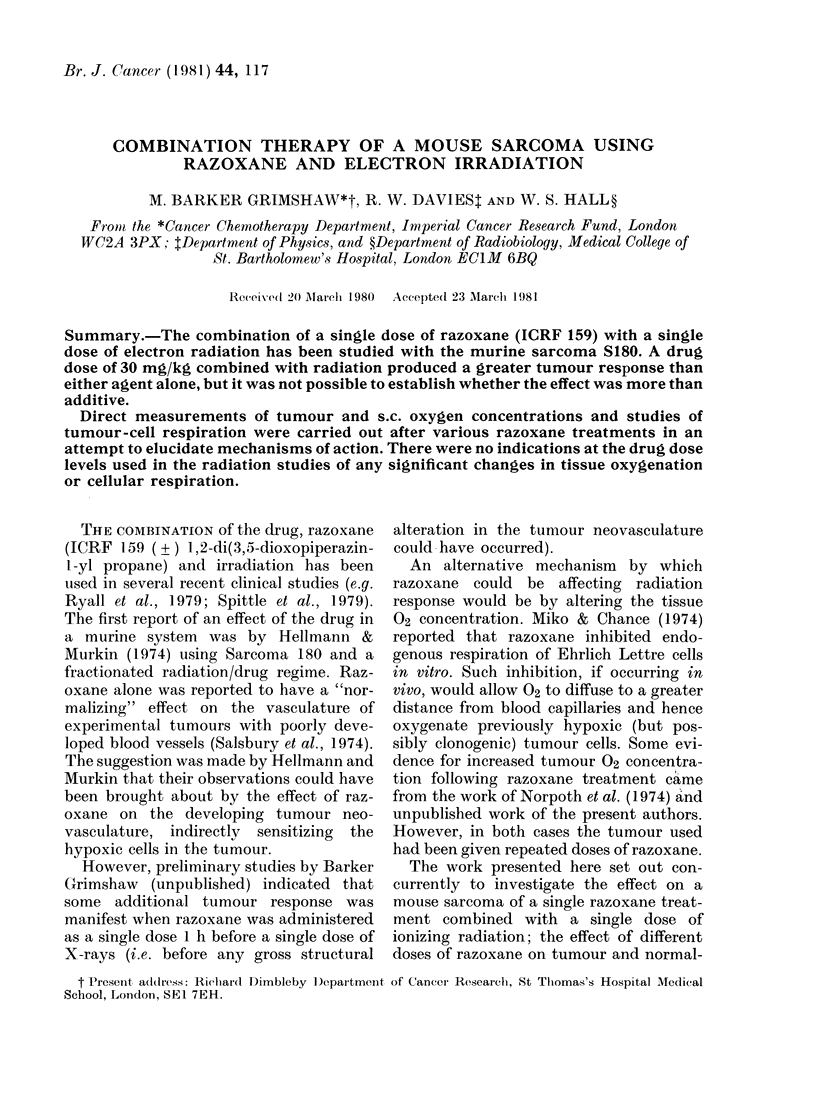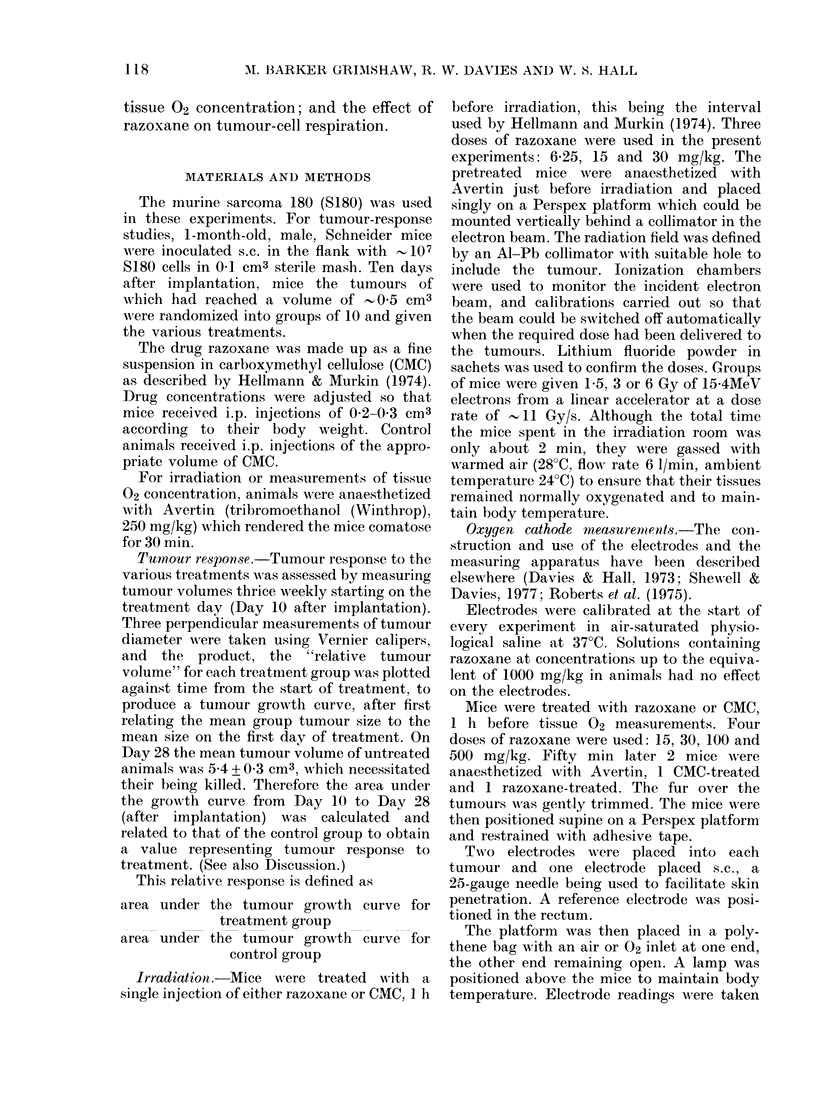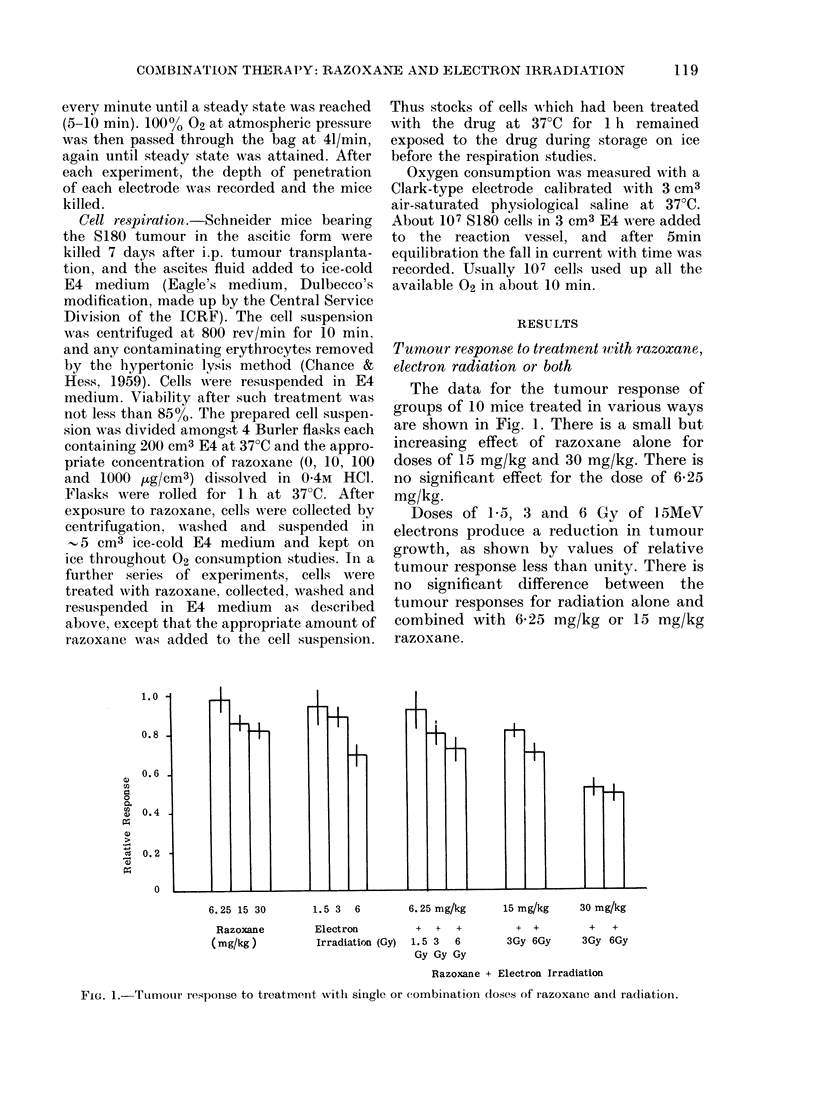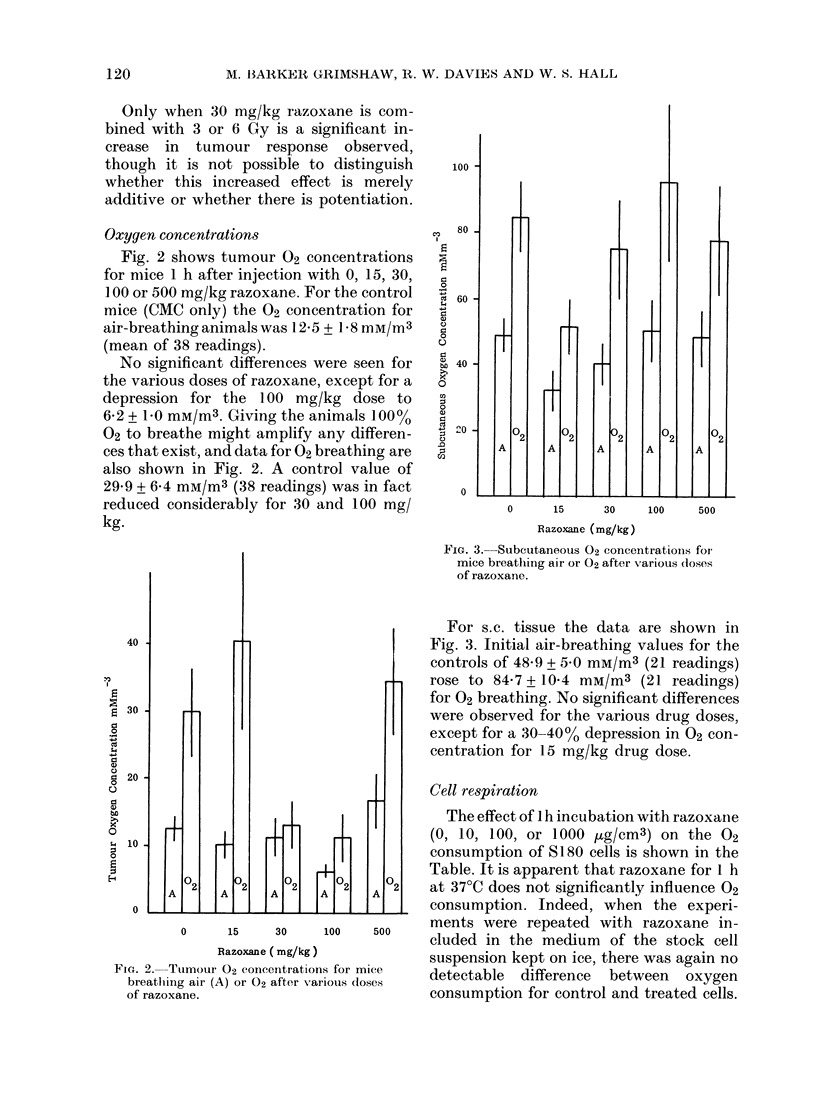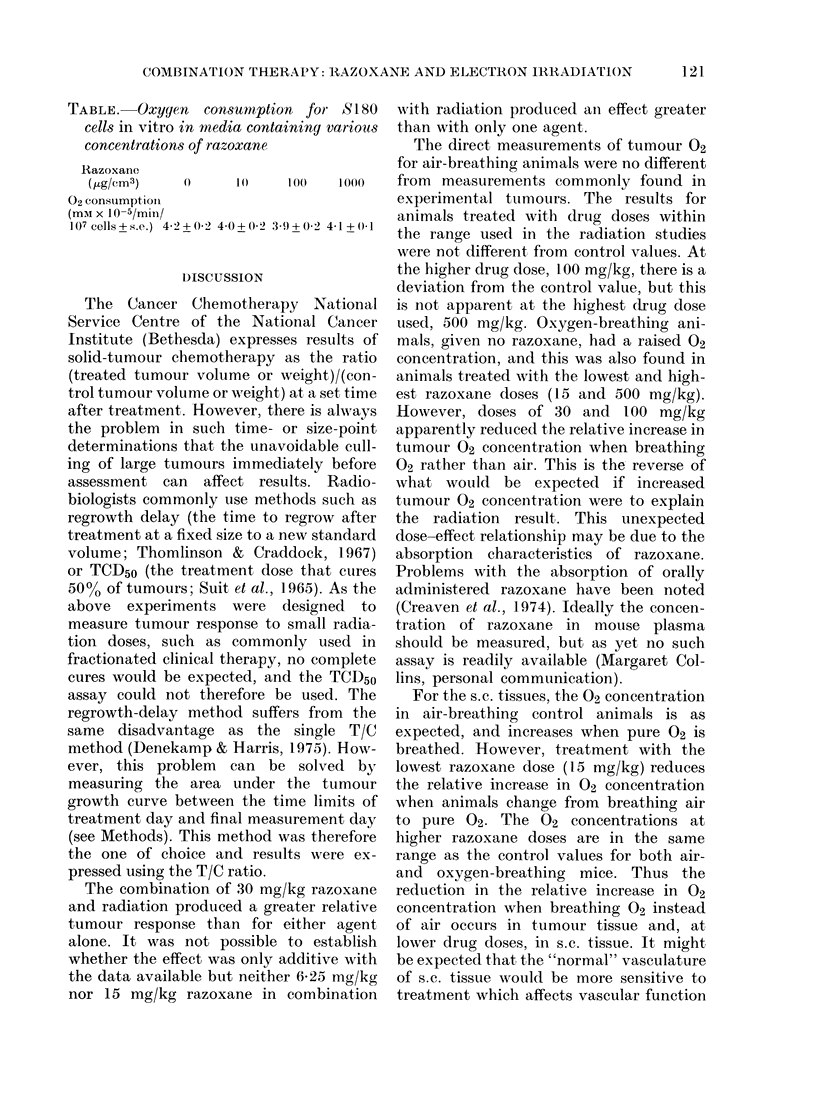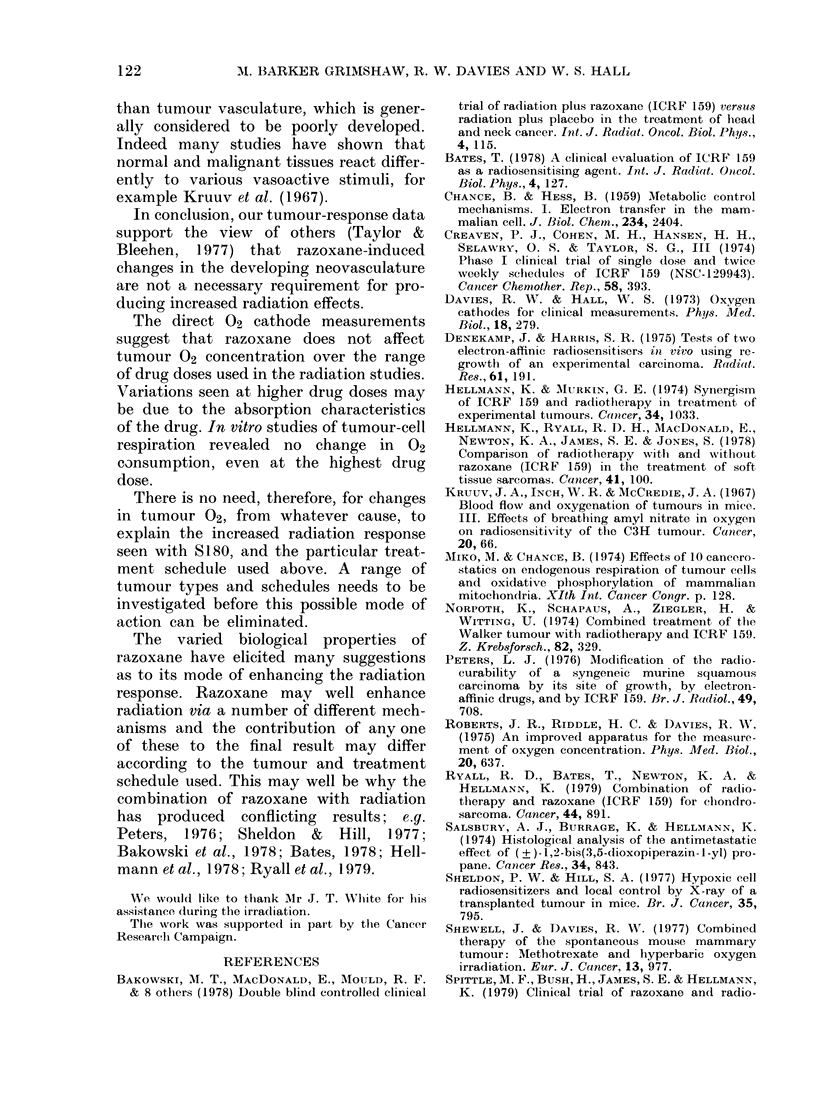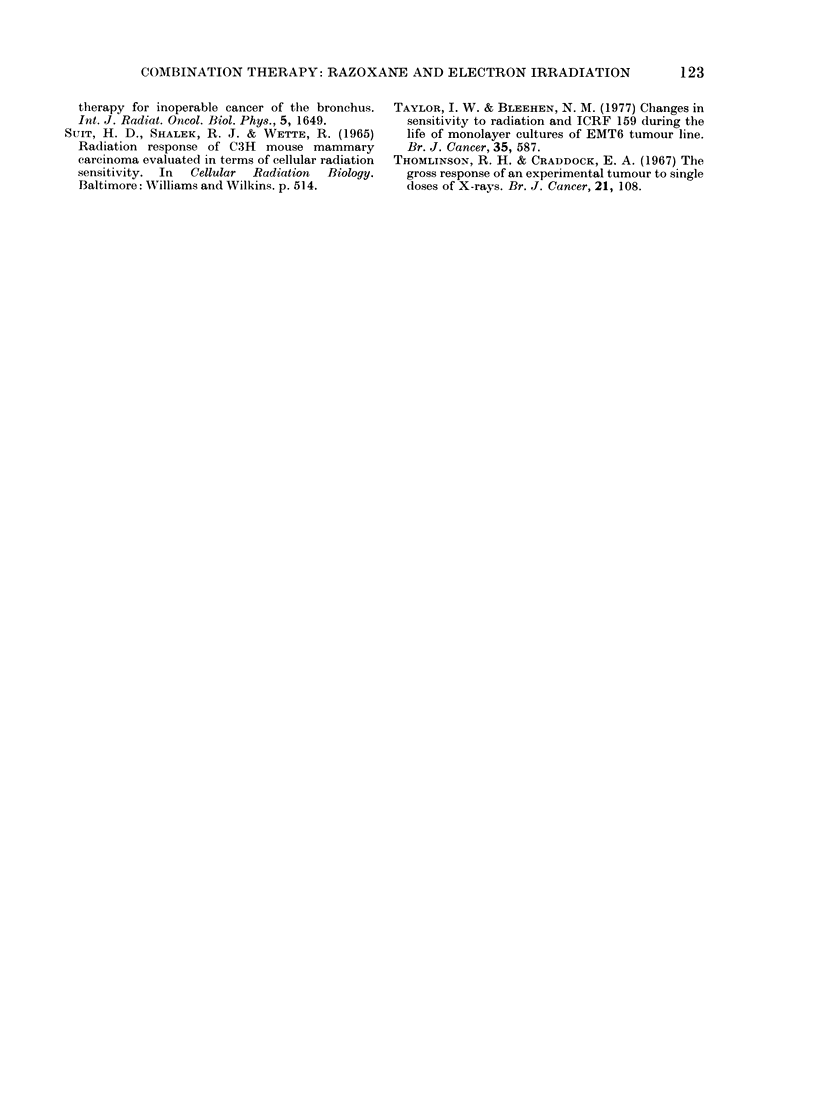# Combination therapy of a mouse sarcoma using razoxane and electron irradiation.

**DOI:** 10.1038/bjc.1981.156

**Published:** 1981-07

**Authors:** M. B. Grimshaw, R. W. Davies, W. S. Hall

## Abstract

The combination of a single dose of razoxane (ICRF 159) with a single dose of electron radiation has been studied with the murine sarcoma S180. A drug dose of 30 mg/kg combined with radiation produced a greater tumour response than either agent alone, but it was not possible to establish whether the effect was more than additive. Direct measurements of tumour and s.c. oxygen concentrations and studies of tumour-cell respiration were carried out after various razoxane treatments in an attempt to elucidate mechanisms of action. There were no indications at the drug dose levels used in the radiation studies of any significant changes in tissue oxygenation or cellular respiration.


*Br. J. Cancer* (1981) **44**, 117

### COMBINATION THERAPY OF A MOUSE SARCOMA USING RAZOXANE AND ELECTRON IRRADIATION

M. BARKER GRIMSHAW*†, R. W. DAVIES‡ AND W. S. HALL§

*From the *Cancer Chemotherapy Department, Imperial Cancer Research Fund, London WC2A 3PX; ‡Department of Physics, and §Department of Radiobiology, Medical College of St. Bartholomew's Hospital, London EC1M 6BQ*

Received 20 March 1980 Accepted 23 March 1981

**Summary.—The combination of a single dose of razoxane (ICRF 159) with a single dose of electron radiation has been studied with the murine sarcoma S180. A drug dose of 30 mg/kg combined with radiation produced a greater tumour response than either agent alone, but it was not possible to establish whether the effect was more than additive.**

**Direct measurements of tumour and s.c. oxygen concentrations and studies of tumour-cell respiration were carried out after various razoxane treatments in an attempt to elucidate mechanisms of action. There were no indications at the drug dose levels used in the radiation studies of any significant changes in tissue oxygenation or cellular respiration.**

THE COMBINATION of the drug, razoxane (ICRF 159 (±) 1,2-di(3,5-dioxopiperazin-1-yl propane) and irradiation has been used in several recent clinical studies (*e.g.* Ryall *et al.*, 1979; Spittle *et al.*, 1979). The first report of an effect of the drug in a murine system was by Hellmann & Murkin (1974) using Sarcoma 180 and a fractionated radiation/drug regime. Razoxane alone was reported to have a "normalizing" effect on the vasculature of experimental tumours with poorly developed blood vessels (Salsbury *et al.*, 1974). The suggestion was made by Hellmann and Murkin that their observations could have been brought about by the effect of razoxane on the developing tumour neovasculature, indirectly sensitizing the hypoxic cells in the tumour.

However, preliminary studies by Barker Grimshaw (unpublished) indicated that some additional tumour response was manifest when razoxane was administered as a single dose 1 h before a single dose of X-rays (*i.e.* before any gross structural alteration in the tumour neovasculature could have occurred).

An alternative mechanism by which razoxane could be affecting radiation response would be by altering the tissue $O_2$ concentration. Miko & Chance (1974) reported that razoxane inhibited endogenous respiration of Ehrlich Lettre cells *in vitro*. Such inhibition, if occurring *in vivo*, would allow $O_2$ to diffuse to a greater distance from blood capillaries and hence oxygenate previously hypoxic (but possibly clonogenic) tumour cells. Some evidence for increased tumour $O_2$ concentration following razoxane treatment came from the work of Norpoth *et al.* (1974) and unpublished work of the present authors. However, in both cases the tumour used had been given repeated doses of razoxane.

The work presented here set out concurrently to investigate the effect on a mouse sarcoma of a single razoxane treatment combined with a single dose of ionizing radiation; the effect of different doses of razoxane on tumour and normal-

† Present address: Richard Dimbleby Department of Cancer Research, St Thomas's Hospital Medical School, London, SE1 7EH.

tissue $O_2$ concentration; and the effect of razoxane on tumour-cell respiration.

### MATERIALS AND METHODS

The murine sarcoma 180 (S180) was used in these experiments. For tumour-response studies, 1-month-old, male, Schneider mice were inoculated s.c. in the flank with $\sim 10^7$ S180 cells in 0·1 $cm^3$ sterile mash. Ten days after implantation, mice the tumours of which had reached a volume of $\sim 0{\cdot}5$ $cm^3$ were randomized into groups of 10 and given the various treatments.

The drug razoxane was made up as a fine suspension in carboxymethyl cellulose (CMC) as described by Hellmann & Murkin (1974). Drug concentrations were adjusted so that mice received i.p. injections of 0·2–0·3 $cm^3$ according to their body weight. Control animals received i.p. injections of the appropriate volume of CMC.

For irradiation or measurements of tissue $O_2$ concentration, animals were anaesthetized with Avertin (tribromoethanol (Winthrop), 250 mg/kg) which rendered the mice comatose for 30 min.

*Tumour response.*—Tumour response to the various treatments was assessed by measuring tumour volumes thrice weekly starting on the treatment day (Day 10 after implantation). Three perpendicular measurements of tumour diameter were taken using Vernier calipers, and the product, the "relative tumour volume" for each treatment group was plotted against time from the start of treatment, to produce a tumour growth curve, after first relating the mean group tumour size to the mean size on the first day of treatment. On Day 28 the mean tumour volume of untreated animals was $5{\cdot}4 \pm 0{\cdot}3$ $cm^3$, which necessitated their being killed. Therefore the area under the growth curve from Day 10 to Day 28 (after implantation) was calculated and related to that of the control group to obtain a value representing tumour response to treatment. (See also Discussion.)

This relative response is defined as

$$\frac{\text{area under the tumour growth curve for treatment group}}{\text{area under the tumour growth curve for control group}}$$

*Irradiation.*—Mice were treated with a single injection of either razoxane or CMC, 1 h before irradiation, this being the interval used by Hellmann and Murkin (1974). Three doses of razoxane were used in the present experiments: 6·25, 15 and 30 mg/kg. The pretreated mice were anaesthetized with Avertin just before irradiation and placed singly on a Perspex platform which could be mounted vertically behind a collimator in the electron beam. The radiation field was defined by an Al–Pb collimator with suitable hole to include the tumour. Ionization chambers were used to monitor the incident electron beam, and calibrations carried out so that the beam could be switched off automatically when the required dose had been delivered to the tumours. Lithium fluoride powder in sachets was used to confirm the doses. Groups of mice were given 1·5, 3 or 6 Gy of 15·4MeV electrons from a linear accelerator at a dose rate of $\sim 11$ Gy/s. Although the total time the mice spent in the irradiation room was only about 2 min, they were gassed with warmed air (28°C, flow rate 6 l/min, ambient temperature 24°C) to ensure that their tissues remained normally oxygenated and to maintain body temperature.

*Oxygen cathode measurements.*—The construction and use of the electrodes and the measuring apparatus have been described elsewhere (Davies & Hall, 1973; Shewell & Davies, 1977; Roberts *et al.* (1975).

Electrodes were calibrated at the start of every experiment in air-saturated physiological saline at 37°C. Solutions containing razoxane at concentrations up to the equivalent of 1000 mg/kg in animals had no effect on the electrodes.

Mice were treated with razoxane or CMC, 1 h before tissue $O_2$ measurements. Four doses of razoxane were used: 15, 30, 100 and 500 mg/kg. Fifty min later 2 mice were anaesthetized with Avertin, 1 CMC-treated and 1 razoxane-treated. The fur over the tumours was gently trimmed. The mice were then positioned supine on a Perspex platform and restrained with adhesive tape.

Two electrodes were placed into each tumour and one electrode placed s.c., a 25-gauge needle being used to facilitate skin penetration. A reference electrode was positioned in the rectum.

The platform was then placed in a polythene bag with an air or $O_2$ inlet at one end, the other end remaining open. A lamp was positioned above the mice to maintain body temperature. Electrode readings were taken every minute until a steady state was reached (5–10 min). 100% $O_2$ at atmospheric pressure was then passed through the bag at 4l/min, again until steady state was attained. After each experiment, the depth of penetration of each electrode was recorded and the mice killed.

*Cell respiration.*—Schneider mice bearing the S180 tumour in the ascitic form were killed 7 days after i.p. tumour transplantation, and the ascites fluid added to ice-cold E4 medium (Eagle's medium, Dulbecco's modification, made up by the Central Service Division of the ICRF). The cell suspension was centrifuged at 800 rev/min for 10 min, and any contaminating erythrocytes removed by the hypertonic lysis method (Chance & Hess, 1959). Cells were resuspended in E4 medium. Viability after such treatment was not less than 85%. The prepared cell suspension was divided amongst 4 Burler flasks each containing 200 cm³ E4 at 37°C and the appropriate concentration of razoxane (0, 10, 100 and 1000 $\mu g/cm^3$) dissolved in 0·4M HCl. Flasks were rolled for 1 h at 37°C. After exposure to razoxane, cells were collected by centrifugation, washed and suspended in ~5 cm³ ice-cold E4 medium and kept on ice throughout $O_2$ consumption studies. In a further series of experiments, cells were treated with razoxane, collected, washed and resuspended in E4 medium as described above, except that the appropriate amount of razoxane was added to the cell suspension. Thus stocks of cells which had been treated with the drug at 37°C for 1 h remained exposed to the drug during storage on ice before the respiration studies.

Oxygen consumption was measured with a Clark-type electrode calibrated with 3 cm³ air-saturated physiological saline at 37°C. About $10^7$ S180 cells in 3 cm³ E4 were added to the reaction vessel, and after 5min equilibration the fall in current with time was recorded. Usually $10^7$ cells used up all the available $O_2$ in about 10 min.

### RESULTS

### *Tumour response to treatment with razoxane, electron radiation or both*

The data for the tumour response of groups of 10 mice treated in various ways are shown in Fig. 1. There is a small but increasing effect of razoxane alone for doses of 15 mg/kg and 30 mg/kg. There is no significant effect for the dose of 6·25 mg/kg.

Doses of 1·5, 3 and 6 Gy of 15MeV electrons produce a reduction in tumour growth, as shown by values of relative tumour response less than unity. There is no significant difference between the tumour responses for radiation alone and combined with 6·25 mg/kg or 15 mg/kg razoxane.

FIG. 1.—Tumour response to treatment with single or combination doses of razoxane and radiation.

Only when 30 mg/kg razoxane is combined with 3 or 6 Gy is a significant increase in tumour response observed, though it is not possible to distinguish whether this increased effect is merely additive or whether there is potentiation.

*Oxygen concentrations*

Fig. 2 shows tumour $O_2$ concentrations for mice 1 h after injection with 0, 15, 30, 100 or 500 mg/kg razoxane. For the control mice (CMC only) the $O_2$ concentration for air-breathing animals was $12{\cdot}5 \pm 1{\cdot}8$ mM/m$^3$ (mean of 38 readings).

No significant differences were seen for the various doses of razoxane, except for a depression for the 100 mg/kg dose to $6{\cdot}2 \pm 1{\cdot}0$ mM/m$^3$. Giving the animals 100% $O_2$ to breathe might amplify any differences that exist, and data for $O_2$ breathing are also shown in Fig. 2. A control value of $29{\cdot}9 \pm 6{\cdot}4$ mM/m$^3$ (38 readings) was in fact reduced considerably for 30 and 100 mg/kg.

FIG. 2.—Tumour $O_2$ concentrations for mice breathing air (A) or $O_2$ after various doses of razoxane.

FIG. 3.—Subcutaneous $O_2$ concentrations for mice breathing air or $O_2$ after various doses of razoxane.

For s.c. tissue the data are shown in Fig. 3. Initial air-breathing values for the controls of $48{\cdot}9 \pm 5{\cdot}0$ mM/m$^3$ (21 readings) rose to $84{\cdot}7 \pm 10{\cdot}4$ mM/m$^3$ (21 readings) for $O_2$ breathing. No significant differences were observed for the various drug doses, except for a 30–40% depression in $O_2$ concentration for 15 mg/kg drug dose.

*Cell respiration*

The effect of 1 h incubation with razoxane (0, 10, 100, or 1000 $\mu$g/cm$^3$) on the $O_2$ consumption of S180 cells is shown in the Table. It is apparent that razoxane for 1 h at 37°C does not significantly influence $O_2$ consumption. Indeed, when the experiments were repeated with razoxane included in the medium of the stock cell suspension kept on ice, there was again no detectable difference between oxygen consumption for control and treated cells.

TABLE.—*Oxygen consumption for S180 cells* in vitro *in media containing various concentrations of razoxane*

| Razoxane ($\mu$g/cm$^3$) | 0 | 10 | 100 | 1000 |
|---|---|---|---|---|
| $O_2$ consumption (mM × $10^{-5}$/min/ $10^7$ cells ± s.e.) | 4·2 ± 0·2 | 4·0 ± 0·2 | 3·9 ± 0·2 | 4·1 ± 0·1 |

### DISCUSSION

The Cancer Chemotherapy National Service Centre of the National Cancer Institute (Bethesda) expresses results of solid-tumour chemotherapy as the ratio (treated tumour volume or weight)/(control tumour volume or weight) at a set time after treatment. However, there is always the problem in such time- or size-point determinations that the unavoidable culling of large tumours immediately before assessment can affect results. Radiobiologists commonly use methods such as regrowth delay (the time to regrow after treatment at a fixed size to a new standard volume; Thomlinson & Craddock, 1967) or $TCD_{50}$ (the treatment dose that cures 50% of tumours; Suit *et al.*, 1965). As the above experiments were designed to measure tumour response to small radiation doses, such as commonly used in fractionated clinical therapy, no complete cures would be expected, and the $TCD_{50}$ assay could not therefore be used. The regrowth-delay method suffers from the same disadvantage as the single T/C method (Denekamp & Harris, 1975). However, this problem can be solved by measuring the area under the tumour growth curve between the time limits of treatment day and final measurement day (see Methods). This method was therefore the one of choice and results were expressed using the T/C ratio.

The combination of 30 mg/kg razoxane and radiation produced a greater relative tumour response than for either agent alone. It was not possible to establish whether the effect was only additive with the data available but neither 6·25 mg/kg nor 15 mg/kg razoxane in combination with radiation produced an effect greater than with only one agent.

The direct measurements of tumour $O_2$ for air-breathing animals were no different from measurements commonly found in experimental tumours. The results for animals treated with drug doses within the range used in the radiation studies were not different from control values. At the higher drug dose, 100 mg/kg, there is a deviation from the control value, but this is not apparent at the highest drug dose used, 500 mg/kg. Oxygen-breathing animals, given no razoxane, had a raised $O_2$ concentration, and this was also found in animals treated with the lowest and highest razoxane doses (15 and 500 mg/kg). However, doses of 30 and 100 mg/kg apparently reduced the relative increase in tumour $O_2$ concentration when breathing $O_2$ rather than air. This is the reverse of what would be expected if increased tumour $O_2$ concentration were to explain the radiation result. This unexpected dose–effect relationship may be due to the absorption characteristics of razoxane. Problems with the absorption of orally administered razoxane have been noted (Creaven *et al.*, 1974). Ideally the concentration of razoxane in mouse plasma should be measured, but as yet no such assay is readily available (Margaret Collins, personal communication).

For the s.c. tissues, the $O_2$ concentration in air-breathing control animals is as expected, and increases when pure $O_2$ is breathed. However, treatment with the lowest razoxane dose (15 mg/kg) reduces the relative increase in $O_2$ concentration when animals change from breathing air to pure $O_2$. The $O_2$ concentrations at higher razoxane doses are in the same range as the control values for both air- and oxygen-breathing mice. Thus the reduction in the relative increase in $O_2$ concentration when breathing $O_2$ instead of air occurs in tumour tissue and, at lower drug doses, in s.c. tissue. It might be expected that the "normal" vasculature of s.c. tissue would be more sensitive to treatment which affects vascular function than tumour vasculature, which is generally considered to be poorly developed. Indeed many studies have shown that normal and malignant tissues react differently to various vasoactive stimuli, for example Kruuv *et al.* (1967).

In conclusion, our tumour-response data support the view of others (Taylor & Bleehen, 1977) that razoxane-induced changes in the developing neovasculature are not a necessary requirement for producing increased radiation effects.

The direct $O_2$ cathode measurements suggest that razoxane does not affect tumour $O_2$ concentration over the range of drug doses used in the radiation studies. Variations seen at higher drug doses may be due to the absorption characteristics of the drug. *In vitro* studies of tumour-cell respiration revealed no change in $O_2$ consumption, even at the highest drug dose.

There is no need, therefore, for changes in tumour $O_2$, from whatever cause, to explain the increased radiation response seen with S180, and the particular treatment schedule used above. A range of tumour types and schedules needs to be investigated before this possible mode of action can be eliminated.

The varied biological properties of razoxane have elicited many suggestions as to its mode of enhancing the radiation response. Razoxane may well enhance radiation *via* a number of different mechanisms and the contribution of any one of these to the final result may differ according to the tumour and treatment schedule used. This may well be why the combination of razoxane with radiation has produced conflicting results; *e.g.* Peters, 1976; Sheldon & Hill, 1977; Bakowski *et al.*, 1978; Bates, 1978; Hellmann *et al.*, 1978; Ryall *et al.*, 1979.

We would like to thank Mr J. T. White for his assistance during the irradiation.

The work was supported in part by the Cancer Research Campaign.

### REFERENCES

Bakowski, M. T., MacDonald, E., Mould, R. F. & 8 others (1978) Double blind controlled clinical trial of radiation plus razoxane (ICRF 159) *versus* radiation plus placebo in the treatment of head and neck cancer. *Int. J. Radiat. Oncol. Biol. Phys.*, **4**, 115.

Bates, T. (1978) A clinical evaluation of ICRF 159 as a radiosensitising agent. *Int. J. Radiat. Oncol. Biol. Phys.*, **4**, 127.

Chance, B. & Hess, B. (1959) Metabolic control mechanisms. I. Electron transfer in the mammalian cell. *J. Biol. Chem.*, **234**, 2404.

Creaven, P. J., Cohen, M. H., Hansen, H. H., Selawry, O. S. & Taylor, S. G., III (1974) Phase I clinical trial of single dose and twice weekly schedules of ICRF 159 (NSC-129943). *Cancer Chemother. Rep.*, **58**, 393.

Davies, R. W. & Hall, W. S. (1973) Oxygen cathodes for clinical measurements. *Phys. Med. Biol.*, **18**, 279.

Denekamp, J. & Harris, S. R. (1975) Tests of two electron-affinic radiosensitisers *in vivo* using regrowth of an experimental carcinoma. *Radiat. Res.*, **61**, 191.

Hellmann, K. & Murkin, G. E. (1974) Synergism of ICRF 159 and radiotherapy in treatment of experimental tumours. *Cancer*, **34**, 1033.

Hellmann, K., Ryall, R. D. H., MacDonald, E., Newton, K. A., James, S. E. & Jones, S. (1978) Comparison of radiotherapy with and without razoxane (ICRF 159) in the treatment of soft tissue sarcomas. *Cancer*, **41**, 100.

Kruuv, J. A., Inch, W. R. & McCredie, J. A. (1967) Blood flow and oxygenation of tumours in mice. III. Effects of breathing amyl nitrate in oxygen on radiosensitivity of the C3H tumour. *Cancer*, **20**, 66.

Miko, M. & Chance, B. (1974) Effects of 10 cancerostatics on endogenous respiration of tumour cells and oxidative phosphorylation of mammalian mitochondria. *XIth Int. Cancer Congr.* p. 128.

Norpoth, K., Schapaus, A., Ziegler, H. & Witting, U. (1974) Combined treatment of the Walker tumour with radiotherapy and ICRF 159. *Z. Krebsforsch.*, **82**, 329.

Peters, L. J. (1976) Modification of the radiocurability of a syngeneic murine squamous carcinoma by its site of growth, by electron-affinic drugs, and by ICRF 159. *Br. J. Radiol.*, **49**, 708.

Roberts, J. R., Riddle, H. C. & Davies, R. W. (1975) An improved apparatus for the measurement of oxygen concentration. *Phys. Med. Biol.*, **20**, 637.

Ryall, R. D., Bates, T., Newton, K. A. & Hellmann, K. (1979) Combination of radiotherapy and razoxane (ICRF 159) for chondrosarcoma. *Cancer*, **44**, 891.

Salsbury, A. J., Burrage, K. & Hellmann, K. (1974) Histological analysis of the antimetastatic effect of (±)-1,2-bis(3,5-dioxopiperazin-1-yl) propane. *Cancer Res.*, **34**, 843.

Sheldon, P. W. & Hill, S. A. (1977) Hypoxic cell radiosensitizers and local control by X-ray of a transplanted tumour in mice. *Br. J. Cancer*, **35**, 795.

Shewell, J. & Davies, R. W. (1977) Combined therapy of the spontaneous mouse mammary tumour: Methotrexate and hyperbaric oxygen irradiation. *Eur. J. Cancer*, **13**, 977.

Spittle, M. F., Bush, H., James, S. E. & Hellmann, K. (1979) Clinical trial of razoxane and radiotherapy for inoperable cancer of the bronchus. *Int. J. Radiat. Oncol. Biol. Phys.*, **5**, 1649.

SUIT, H. D., SHALEK, R. J. & WETTE, R. (1965) Radiation response of C3H mouse mammary carcinoma evaluated in terms of cellular radiation sensitivity. In *Cellular Radiation Biology*. Baltimore: Williams and Wilkins. p. 514.

TAYLOR, I. W. & BLEEHEN, N. M. (1977) Changes in sensitivity to radiation and ICRF 159 during the life of monolayer cultures of EMT6 tumour line. *Br. J. Cancer*, **35**, 587.

THOMLINSON, R. H. & CRADDOCK, E. A. (1967) The gross response of an experimental tumour to single doses of X-rays. *Br. J. Cancer*, **21**, 108.